# Current Status of *Pseudaulacaspis pentagona* (Targioni-Tozzetti): Context, Impact and Challenges for Integrated Management in Ecuador

**DOI:** 10.3390/insects17080758

**Published:** 2026-07-24

**Authors:** Telmo-Fernando Basantes-Vizcaino, Luis Marcelo Albuja-Illescas, Julia K. Prado, Bolívar Xavier Aguirre Valencia

**Affiliations:** 1 Agrobiodiversity and Food Security Research Group—GIASSA, Agricultural and Environmental Science Faculty, Universidad Técnica del Norte, Av. 17 de Julio 5-21 y Gral. José María Córdova, Ibarra EC100105, Ecuador; lmalbuja@utn.edu.ec (L.M.A.-I.); jkprado@utn.edu.ec (J.K.P.); 2 Agency for Phytosanitary and Zoosanitary Regulation and Control (AGROCALIDAD), Calle Olmedo 1086 y Chimborazo, Tulcán 040103, Ecuador; xavier.aguirre@agrocalidad.gob.ec

**Keywords:** *Pseudaulacaspis pentagona*, integrated pest management, quarantine risk, Andean fruit production, biological control, Ecuador

## Abstract

The white peach scale, *Pseudaulacaspis pentagona* (Targioni-Tozzetti) (Hemiptera: Diaspididae), is a highly polyphagous insect pest of global phytosanitary relevance affecting fruit trees, perennial crops, and ornamental plants in temperate and subtropical regions. Its invasive success is largely driven by high thermal tolerance, a wide host range, and strong survival capacity under postharvest handling and cold-chain storage, which greatly enhances its potential for long-distance dispersal through international trade of fresh fruit and plant material. A systematic review and meta-synthesis of 105 scientific studies published between 1958 and 2026 identified key research areas, including geographic distribution and invasion pathways, host plants and varietal susceptibility, temperature-dependent life history, agricultural impact and quarantine risk, chemical and biological control strategies, and the development of phenology-based monitoring and prediction tools using thermal models and pheromones. Although recent official reports do not confirm the presence of *P. pentagona* in Ecuador, climate suitability modeling and evidence of long-term survival during cold storage indicate a high risk of introduction and establishment, particularly in inter-Andean valleys. Consequently, preventive phytosanitary surveillance and integrated pest management strategies tailored to Ecuadorian Andean fruit production are proposed, emphasizing phenological monitoring, nursery and agro-urban inspections, and the conservation of natural enemies as a foundation for sustainable management of this emerging pest.

## Simple Summary

The white peach scale, *Pseudaulacaspis pentagona*, is an invasive insect that attacks more than 200 plant species, including fruit trees and ornamental plants, causing significant economic losses worldwide. Its ability to survive under a wide range of temperatures and during cold storage increases the risk of spreading through international trade. This review summarizes nearly 70 years of research on the pest, covering its distribution, host plants, biology, economic importance, and available management strategies. Although *P. pentagona* has not been officially reported in Ecuador, climate conditions in several Andean fruit-growing areas could favor its establishment if introduced. The findings highlight the need for preventive phytosanitary surveillance, early detection programs, and integrated pest management strategies that combine field monitoring, biological control, and regular inspections of nurseries and imported plant material. These actions will help protect Ecuadorian fruit production, reduce the risk of pest establishment, and support sustainable agricultural systems.

## 1. Introduction

Diaspididae (Hemiptera: Coccomorpha), commonly known as armored scale insects, constitute one of the most diverse and economically important families of phytophagous insects with an obligate parasitic habit. Globally, more than 2700 species have been described, many of which infest agricultural, forestry, and ornamental crops, causing direct economic losses and phytosanitary restrictions on international trade. In particular, at least 116 diaspidid species have been reported infesting citrus species and hybrids, reflecting the high adaptive capacity and broad host range characteristic of this group [1].

**Figure 1:**
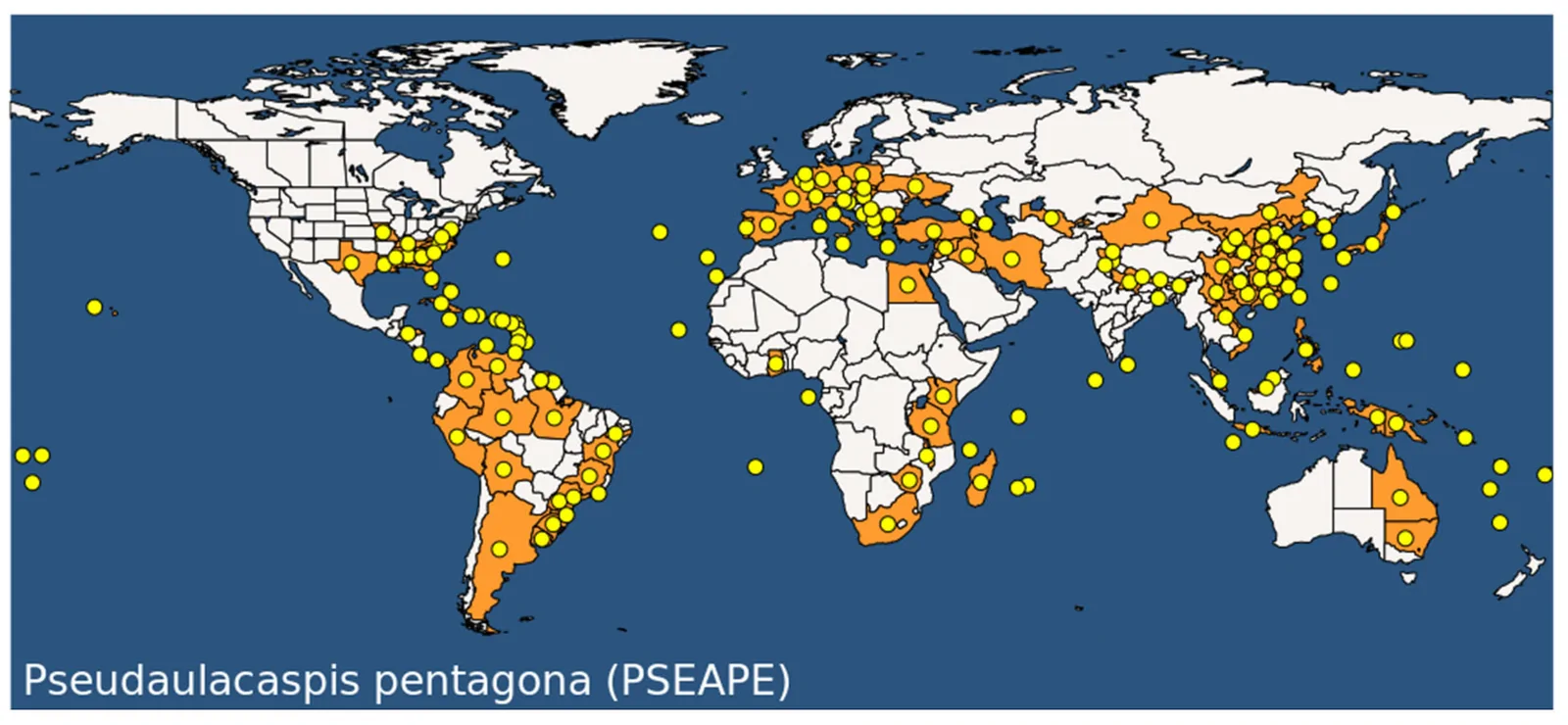
Global distribution of white peach scale (WPS), *Pseudaulacaspis pentagona*, according to EPPO (2026) https://gd.eppo.int (accessed on 8 April 2026) [2]. Yellow dots: Countries with the presence of *P. pentagona*.

Within this family, *Pseudaulacaspis pentagona* (Targioni-Tozzetti), known as the white peach scale, has become established as an invasive pest of global importance due to its pronounced polyphagy, high ecological plasticity, and ability to establish in diverse production systems. Its wide geographic distribution and the diversity of environments it colonizes increase the potential risk for countries where its presence has not yet been officially confirmed. In this context, Ecuador is of relevance, as *P. pentagona* is not currently recorded as present in the national territory (Figure 1), reinforcing its status as a quarantine pest and highlighting the need for a comprehensive assessment of the risk it poses to economically important perennial crops European and Mediterranean Plant Protection Organization [2]. Early and accurate identification of this species is critical for its potential detection and for the timely implementation of management and control strategies, especially under scenarios of increasing trade in plant material and horticultural products [3].

In Ecuador, phytosanitary epidemiological surveillance of quarantine pests constitutes a strategic component of the national agricultural health system, aimed at the prevention, early detection, control, and, when necessary, eradication of organisms that threaten agricultural production, food security, and access to national and international markets. Within this framework, *Pseudaulacaspis pentagona* represents a potential risk that must be addressed in accordance with current national regulations, under the authority of the Agency for Phytosanitary and Zoosanitary Regulation and Control [4], and aligned with the guidelines of the International Plant Protection Convention (IPPC) [5] and the international standards promoted by FAO [6].

Several perennial crops of relevance to Ecuadorian fruit production, such as Prunus persica L., *Persea americana* Mill., and Annona muricata, have been identified in international literature as potential hosts of *P. pentagona*. This species feeds on sap from branches, trunks, and, in some cases, the root system, causing progressive weakening of the plant and, under high population densities, branch dieback or even the death of entire plants, particularly in peach and other woody fruit trees [7]. However, damage caused by this scale insect is mostly described qualitatively as reduced vigor, physiological decline, or severe injury, or through generalized estimates of economic losses. There is no standardized percentage of yield loss attributable exclusively to this pest in perennial crops, which limits comparisons among production systems and regions [8].

Despite this limitation, clear evidence of its economic impact exists. In unmanaged Pyrus communis orchards in the United States, *P. pentagona* has been associated with annual economic losses of approximately USD 480,000 [9]. Similarly, in Hawaii, severe infestations in Carica papaya have led to significant quarantine restrictions, highlighting the role of this species as a barrier to international agricultural trade [10,11]. Nymphs of *P. pentagona* tend to establish initially in protected parts of the host, including roots in some forest species, subsequently forming compact colonies that expand from the base of the trunk toward branches and foliage [12,13]. This colonization pattern causes generalized physiological weakening, promotes bark fissuring, and, in advanced cases, can lead to host death, with impacts extending beyond agriculture to forest and ornamental ecosystems [9].

Although technical datasheets, phytosanitary reports, and general reviews of *Pseudaulacaspis pentagona* (Targioni-Tozzetti) document its biology, host range, and control methods, significant gaps remain in the systematic integration of knowledge, particularly in tropical highland regions. In this context, the present review expands the existing literature by providing a systematic mapping of research on this species and a specific application focused on the Andes, where ecological, altitudinal, and productive dynamics create unique pest–host interaction scenarios. This approach not only synthesizes the current state of knowledge but also identifies trends, gaps, and opportunities for sustainable management in Andean agricultural systems. The structure of this review comprises: (i) a systematic mapping of the available scientific literature; (ii) a synthesis of global knowledge about the species; (iii) the application of this evidence to evaluate risks and propose integrated pest management (IPM) strategies and policy recommendations for Andean fruit production systems in Ecuador.

## 2. Methodology of the Systematic Review and Meta-Synthesis

Sources and scope: A total of 105 unique peer-reviewed studies were included in the systematic review and meta-synthesis and subsequently analyzed [12,13,14,15,16,17,18,19,20,21,22,23,24,25,26,27,28,29,30,31,32,33,34,35,36,37,38,39,40,41,42,43,44,45,46,47,48,49,50,51,52,53,54,55,56,57,58,59,60,61,62,63,64,65,66,67,68,69,70,71,72,73,74,75,76,77,78,79,80,81,82,83,84,85,86,87,88,89,90,91,92,93,94,95,96,97,98,99,100,101,102,103,104,105,106,107,108,109,110,111,112,113,114,115,116,117]. The complete list and main characteristics of all included studies are provided in Table A1.

### 2.1. Search Strategy and Databases

A systematic literature search was conducted to identify relevant scientific publications on the biology, ecology, distribution, and management of *Pseudaulacaspis pentagona*. The search was performed on 15 October 2025, using six major scientific databases: SCOPUS, Web of Science (WoS), CityU University of Hong Kong (Dongguan), J-STAGE, CyberLeninka, and the FAO AGRIS International System for Agricultural Science and Technology. The basic search string applied to titles, abstracts, and keywords was:

(“Pseudaulacaspis pentagona (Targioni)” OR “white peach scale” OR “mulberry scale”) AND (biology OR ecology OR distribution OR “host plants” OR management OR control OR invasion OR risk).

The search was restricted to publications published between 1958 and 2026.

### 2.2. Inclusion and Exclusion Criteria

To ensure the relevance and quality of the synthesized data, articles were selected according to predefined criteria.

Inclusion criteria: (1) Original peer-reviewed research articles and comprehensive review papers; (2) studies directly focused on the biology, phenology, geographic distribution, impact, or management of *P. pentagona*; (3) publications issued between 1958 and 2026; and (4) open-access articles published in English, German, Russian, Chinese, or Japanese.

Exclusion criteria: (1) Conference papers and abstracts without full-text availability or requiring payment for access; (2) non-peer-reviewed reports or scientific outreach articles; (3) studies in which *P. pentagona* was mentioned only tangentially without providing species-specific data; and (4) duplicate records identified across different databases.

### 2.3. Data Extraction and Thematic Categorization

Data from the selected studies (n = 105) were extracted into a standardized spreadsheet. Each study was assigned to one or more thematic categories according to its research objectives and principal findings (e.g., studies projecting future ecological niches were classified under “Prediction Models”). Because some comprehensive studies ad-dressed multiple aspects of the species, the categories were not mutually exclusive. This classification approach facilitated the organization and synthesis of the main research themes identified across the reviewed literature.

Similarly, according to their control mechanisms, studies that explicitly evaluated or discussed pest management strategies were categorized based on the specific tactic assessed (e.g., biological, chemical, or cultural control), as described later in the manuscript.

**Figure 2:**
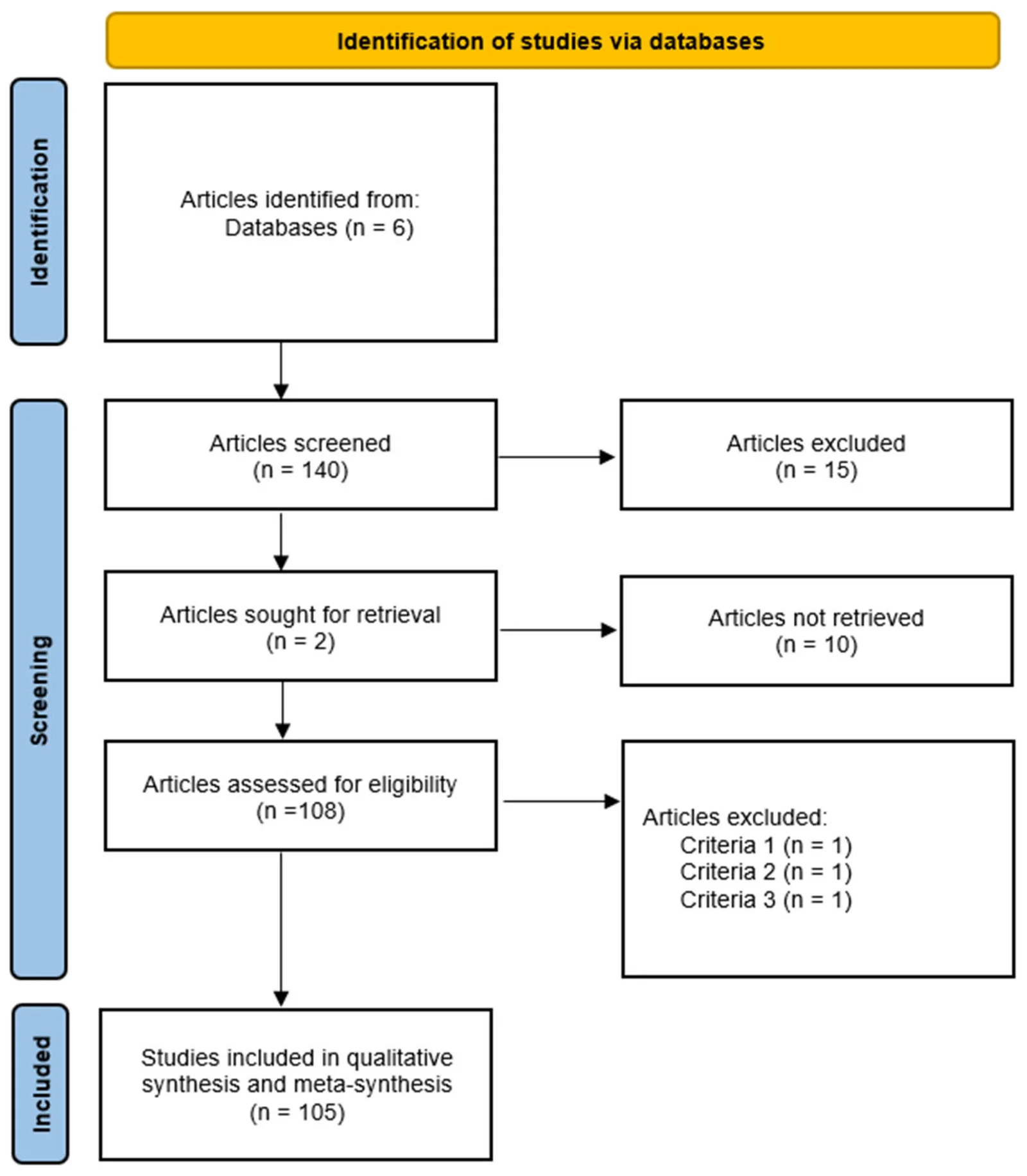
PRISMA flowchart illustrating the study selection process for *P. pentagona.*

In addition, a PRISMA-style flow diagram (Figure 2) was prepared to illustrate the literature selection process, including the records identified, screened, excluded, and ultimately included in the review. This figure provides a concise overview of the screening procedure and enhances the transparency and reproducibility of the study.

It is important to note that the quantitative results presented in this review were derived from the systematic coding of the 105 articles included in the study. In addition, the review incorporates a narrative synthesis that integrates evidence from the scientific literature with agroecological and regulatory information from Ecuador provided by the Ecuadorian Agency for Phytosanitary and Zoosanitary Regulation and Control [4].

**Thematic classification results:**

Table 1 presents the main research categories addressed by the reviewed articles. Several studies covered more than one thematic category; therefore, percentages are not mutually exclusive and sum to more than 100%.

Recent literature focuses on distribution/invasions (29.5%) and predictive models (24.4%; phenology using degree-days, niche/MaxEnt modeling, climate scenarios), followed by biology/life cycle (14.1%) and host plants (11.5%). Critical areas that are less covered in the literature include economic impact and chemical/biological control (each 6.4%), as well as IPM (very scarce when defined as an exclusive category), reinforcing the need for integrated protocols and local validation.

Table 2, in turn, indicates the countries with the highest number of publications in this field.

The countries leading scientific output are Iran and Turkey, followed by the United States, Japan, Italy, Germany, and China. Reports from Latin America are scarce (Brazil, Puerto Rico), with a clear gap in the Andean region, which underscores the relevance of conducting studies in Ecuador.

Regarding the most frequently studied host crops, Table 3 details those mentioned in the publications. It should likewise be noted that several studies reported more than one host; therefore, the percentages are not mutually exclusive, and their sum exceeds 100%.

The most frequently cited systems are *Prunus* (peach and related species), *Morus* (mulberry), *Actinidia* (kiwifruit), and *Camellia sinensis* (tea), along with ornamentals as agro-urban reservoirs; papaya also appears, with quarantine implications.

Additionally, an analysis was conducted of hosts present in Ecuador (Table A2) that could serve as sources of infestation for *P. pentagona* in the inter-Andean valleys.

#### 2.3.1. Control Strategies Reported in the Literature

With respect to the types of control mentioned in the reviewed publications, Table 4 highlights the following:

Mentions of biological control (e.g., *Encarsia*, *Aphytis*, coccinellids) and chemical control (oils, buprofezin) predominate, with monitoring (pheromones, sampling) and IPM as cross-cutting approaches; however, these are less developed in terms of long-term integrated trials.

#### 2.3.2. Temporal Distribution of Studies

Historically, articles have been identified from the 1990s to the present, as classified in Table 5.

A clear increase is observed from 2010 onward, with strong continuity during 2020–2025, particularly in studies on phenology under climate change, varietal resistance (tea/kiwi), and biological control/selective chemical use, consolidating a technically robust agenda suitable for transfer to the Andean context.

## 3. Global Evidence on *Pseudaulacaspis pentagona*

### 3.1. Description, Taxonomy, and Biology of the Insect

Specific local information on the biology, distribution, and economic damage thresholds of *Pseudaulacaspis pentagona* in Ecuador is currently unavailable. This lack of data limits the development of evidence-based management strategies and highlights the need to rely on international literature to assess its potential risk and guide preventive actions.

The white peach scale, *Pseudaulacaspis pentagona* (Targioni-Tozzetti, 1886) (Hemiptera: Diaspididae), is an armored scale insect that has become established as an invasive pest of major phytosanitary importance in fruit, forest, and ornamental systems worldwide. Its invasive success is attributed to a combination of extreme polyphagy, with the ability to colonize more than two hundred plant genera, marked phenotypic plasticity, and temperature-dependent development that allows synchronization of its phenology with temperate and subtropical climates [93,94]. In regions where the species is established, severe damage to woody organs, progressive branch weakening, defoliation, and commercial depreciation of fruit—often associated with localized blemishes at the peduncle insertion point—have been reported, resulting in cumulative negative effects on crop vigor and productivity.

The biology of *P. pentagona* is strongly influenced by temperature and host plant. Life-table studies have demonstrated significant variation in fecundity, survival, and duration of nymphal stages when the insect develops on Morus alba, Morus nigra, Prunus persica, Actinidia deliciosa, and other woody hosts. Lower developmental thresholds generally range between 10.5 and 12.5 °C, and thermal constants expressed in degree-days have been estimated, allowing accurate prediction of crawler emergence—the most vulnerable stage for control [95,96,97,98].

The species exhibits reproductive diapause in overwintering females, regulated primarily by temperature rather than photoperiod. Female sex-pheromone emission, as well as male emergence and flight activity, follow defined circadian rhythms conditioned by temperature and light intensity, directly influencing population dynamics and reproductive success [99,100,101]. These phenological traits have been widely exploited in the development of monitoring and population prediction systems across different agroecosystems.

From a biogeographic perspective, consolidated international databases such as EPPO and GBIF document an almost cosmopolitan distribution of *P. pentagona* across Asia, Europe, Africa, Oceania, and the Americas, with multiple records in South American countries. Although Ecuador is not listed among countries with confirmed presence in the most recent databases, regional climatic suitability and plant-material trade routes increase national vulnerability to potential introduction and establishment [102]. Global ecological niche and potential distribution models, largely based on MaxEnt algorithms, project an expansion of the species’ geographic range under climate-warming scenarios, with direct implications for mountain agricultural systems where temperate and subtropical microclimates coexist. Under such scenarios, an increase in the number of annual generations is expected, modifying infestation pressure and optimal control windows [9,99].

The relevance of *P. pentagona* to Andean fruit production in Ecuador lies in the combination of mesothermal climates in inter-Andean valleys, complex agro-urban landscapes, and the presence of ornamental and fruit nursery supply chains, where urban trees and ornamental plants may act as permanent reservoirs, facilitating reinfestation of productive areas [2,94]. In response to this emerging risk, integrated management of *P. pentagona* should combine complementary strategies, including: (i) phenological monitoring based on degree-day thermal models to detect crawler emergence; (ii) cultural practices such as sanitary pruning, sanitation, and quarantine of propagation material; (iii) strategic use of selective insecticides, such as buprofezin, to minimize negative impacts on natural enemies; and (iv) conservative or augmentative biological control using parasitoids (*Encarsia berlesei*, *Aphytis* spp.) and predators (*Chilocorus* spp., *Rhyzobius lophantae*), integrated within training programs and phytosanitary surveillance schemes [103,104,105,106].

Additionally, recent advances in host-plant resistance research—particularly in kiwifruit—highlight the importance of anatomical traits such as thicker cuticles and higher cortical tissue density, as well as differential activation of jasmonate- and salicylic acid-mediated hormonal pathways associated with reduced susceptibility to infestation. Extrapolating these findings to Andean fruit crops, including peach, plum, and avocado, requires local research and field validation [107].

In Ecuador, key knowledge gaps include the absence of studies quantifying yield and quality losses attributable specifically to *P. pentagona*, the lack of local calibration of phenological and potential distribution models, and the need to develop standardized integrated management protocols adapted to Andean microclimates and smallholder farming systems. Strengthening accurate taxonomic identification through the combined use of morphological characters and molecular markers, such as the COI gene, is also a priority to avoid confusion with closely related *Pseudaulacaspis* species—particularly *P. prunicola*—in landscapes with high diaspidid diversity [108]. In this context, the present article synthesizes the state of the art on the biology, ecology, impacts, and management strategies of *P. pentagona*, and proposes operational guidelines for surveillance and rapid response in Ecuadorian Andean fruit production, prioritizing sustainability and the conservation of beneficial entomofauna [109,110,111].

### 3.2. Life Cycle

*Pseudaulacaspis pentagona* exhibits a multivoltine life cycle, with 2 to 4 generations per year depending on geographic region, altitude, local thermal conditions, and host plant. Adult female fecundity generally ranges from 70 to 150 eggs per female, showing high plasticity in response to temperature and host type, which contributes to the population variability observed among agroecosystems [112,113].

The species is markedly thermophilic, with lower developmental thresholds estimated between 10.5 and 12.5 °C, and thermal constants expressed as degree-days that allow accurate prediction of crawler (mobile nymph) emergence. This information has been widely used to optimize control timing, as crawlers represent the most susceptible stage to chemical and biological interventions [96,97,98,99]. Reproductive diapause in overwintering females is generally maintained at temperatures of 20–22.5 °C and is interrupted near 25 °C, regulating the onset of oviposition and the synchronization of generations [96,97].

**Eggs**

Adult females oviposit beneath their protective waxy scale, providing eggs with high protection against predators, parasitoids, and adverse environmental conditions. Eggs are whitish to yellowish in color, and their development is strongly temperature-dependent. Oviposition rate and egg hatch increase significantly at temperatures above 27–29 °C, although persistently high temperatures may negatively affect embryonic survival [98].

**Nymphs**

**Figure 3:**
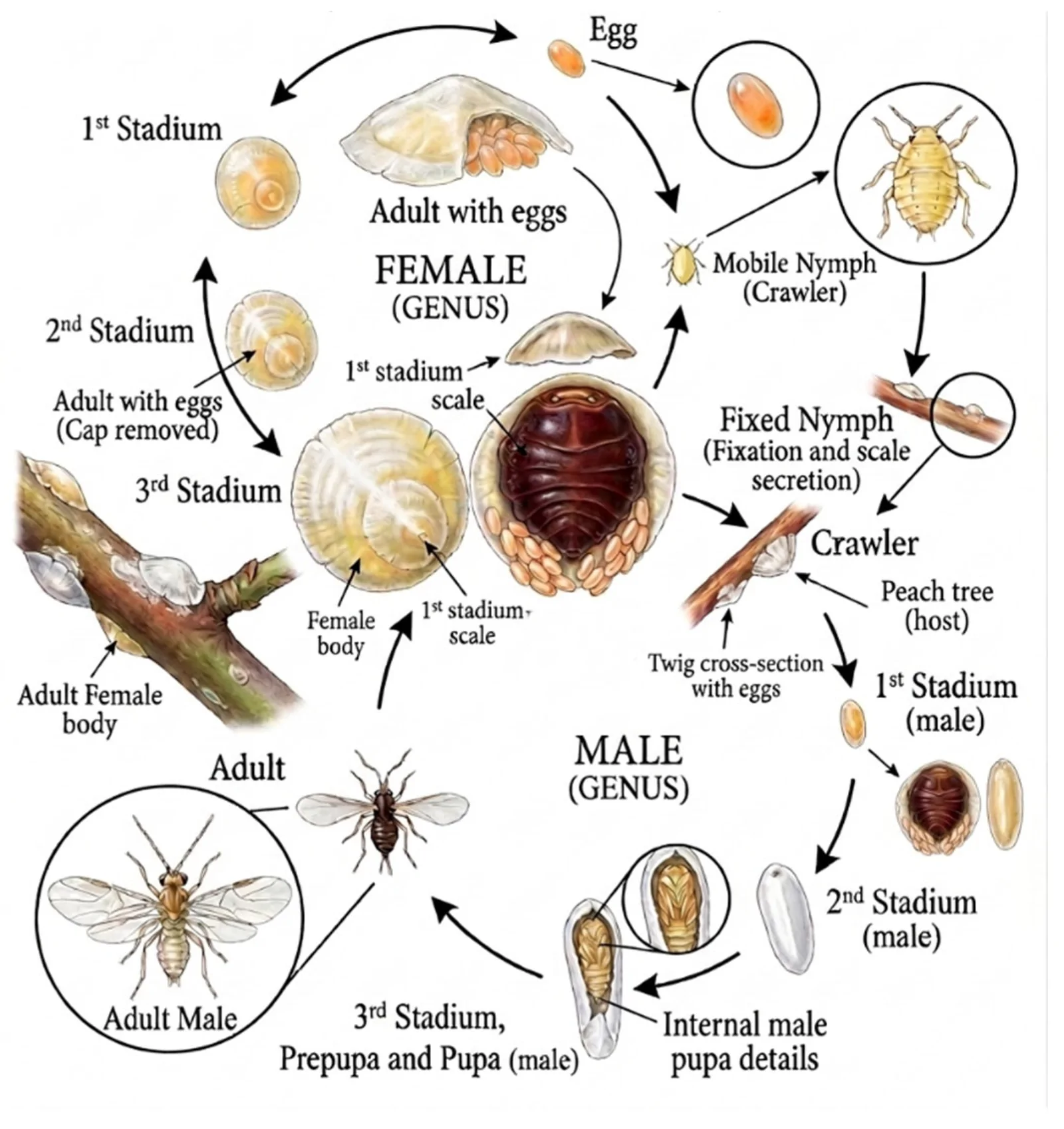
Life cycle of the white peach scale (WPS), *Pseudaulacaspis pentagona* (Modified from [115]. The arrow indicates the sequential progression of the life cycle of *Pseudaulacaspis pentagona*, from the egg stage to the adult stage. The arrow indicates the sequential progression of the life cycle of Pseudaulacaspis pentagona, from the egg stage to the adult stage.

Upon hatching, first-instar mobile nymphs (“crawlers”) emerge and are responsible for primary dispersal within the host plant and among adjacent plants. Crawlers move actively for a short period before settling on woody or semi-woody tissues to initiate feeding. They then develop into sessile nymphal stages, progressively forming the characteristic armored scale of diaspidids (Figure 3) and remain immobile for the remainder of their development [114]. The duration of nymphal stages varies with temperature and host plant, directly influencing population growth rate.

**Adults**

Adult females are wingless, oval-shaped, permanently sessile, and covered by a well-developed white waxy scale. Under controlled conditions, females may oviposit 93 to 133 eggs, although fecundity varies with host plant and thermal regime [113]. Adult males are smaller, winged, and short-lived; their development includes prepupal and pupal stages, and their primary function is mating, with no direct role in feeding [112]. Male emergence and flight follow well-defined temporal patterns controlled by temperature and light, while female sex-pheromone release occurs during the photophase and ceases at the onset of scotophase, resulting in temporal separation between pheromone emission and male activity [99,100,101].

### 3.3. Geographic Distribution and Climate Change

Since its initial identification in East Asia, particularly in China and Japan, *Pseudaulacaspis pentagona* has progressively expanded worldwide, becoming established in regions with intensive agricultural systems and affecting fruit trees, ornamental plants, and woody crops of high economic importance, such as peach, mulberry, and kiwifruit [93,113]. This expansion process has been facilitated by the species’ high ecological adaptability, broad host range, and frequent association with the movement of infested plant material.

Potential distribution analyses indicate that *P. pentagona* shows a strong affinity for temperate to subtropical climates characterized by moderate temperatures, relatively high humidity, and the availability of woody host plants. In Asia, regions identified as highly suitable for establishment include eastern Shandong Peninsula, southwestern China, the coastal areas of Taiwan, northeastern Vietnam, as well as parts of southern Iran, northern Oman, and northern India, where thermal and agroecological conditions favor continuous population development [108].

International phytosanitary databases widely used for pest distribution tracking, such as the EPPO Global Database and the Global Biodiversity Information Facility (GBIF), document a nearly cosmopolitan distribution of *P. pentagona* across Asia, Europe, Africa, Oceania, and the Americas, with numerous confirmed records in North, Central, and South America. However, Ecuador does not appear as a country with confirmed presence in the most recent listings, reinforcing its status as a quarantine pest and highlighting the importance of preventive risk assessment [2,102].

According to historical records compiled by CABI, reported distributions of *P. pentagona* in South America are mainly limited to countries such as Colombia and Peru, with no published evidence confirming its establishment in Ecuador [116]. Nevertheless, when these geographic patterns are contrasted with Ecuadorian agroclimatic conditions, several areas—particularly the northern inter-Andean valleys (Imbabura, Carchi, and Pichincha) and certain coastal zones—exhibit conditions highly comparable to those of regions already affected. The presence of economically important perennial and semi-perennial crops, such as avocado, stone and pome fruits, and forest species, together with relatively stable thermal regimes throughout much of the year, may favor pest establishment and persistence in the absence of timely surveillance and management measures [4].

Multiple studies agree that the geographic expansion of *P. pentagona* is closely associated with the movement of infested plant material, international trade in plants and fruits, and production systems with limited implementation of integrated pest management programs. In these contexts, ornamental nurseries, agro-urban landscapes, and peri-urban areas can act as initial introduction points and sources of secondary dispersal [95]. Consequently, Ecuador faces the challenge of strengthening its prevention, early detection, and phytosanitary control systems, given that climatic similarities with affected regions increase the probability of entry and establishment of this quarantine pest.

Climate change represents an additional risk factor. Ecological niche models based on MaxEnt, together with regional climate projection studies, indicate a potential expansion of the suitable range of *P. pentagona* under global warming scenarios, particularly under high-emission pathways. These models project not only an enlargement of climatically suitable areas, but also an increase in the number of annual generations, which could intensify infestation pressure and modify optimal intervention windows—especially in mountainous agricultural systems and Andean regions [2,9,98]. In this context, integrating climate models, phenological information, and surveillance strategies is essential to anticipate risk scenarios and design phytosanitary responses adapted to local conditions.

### 3.4. Agronomic and Ecological Impacts

**Figure 4:**
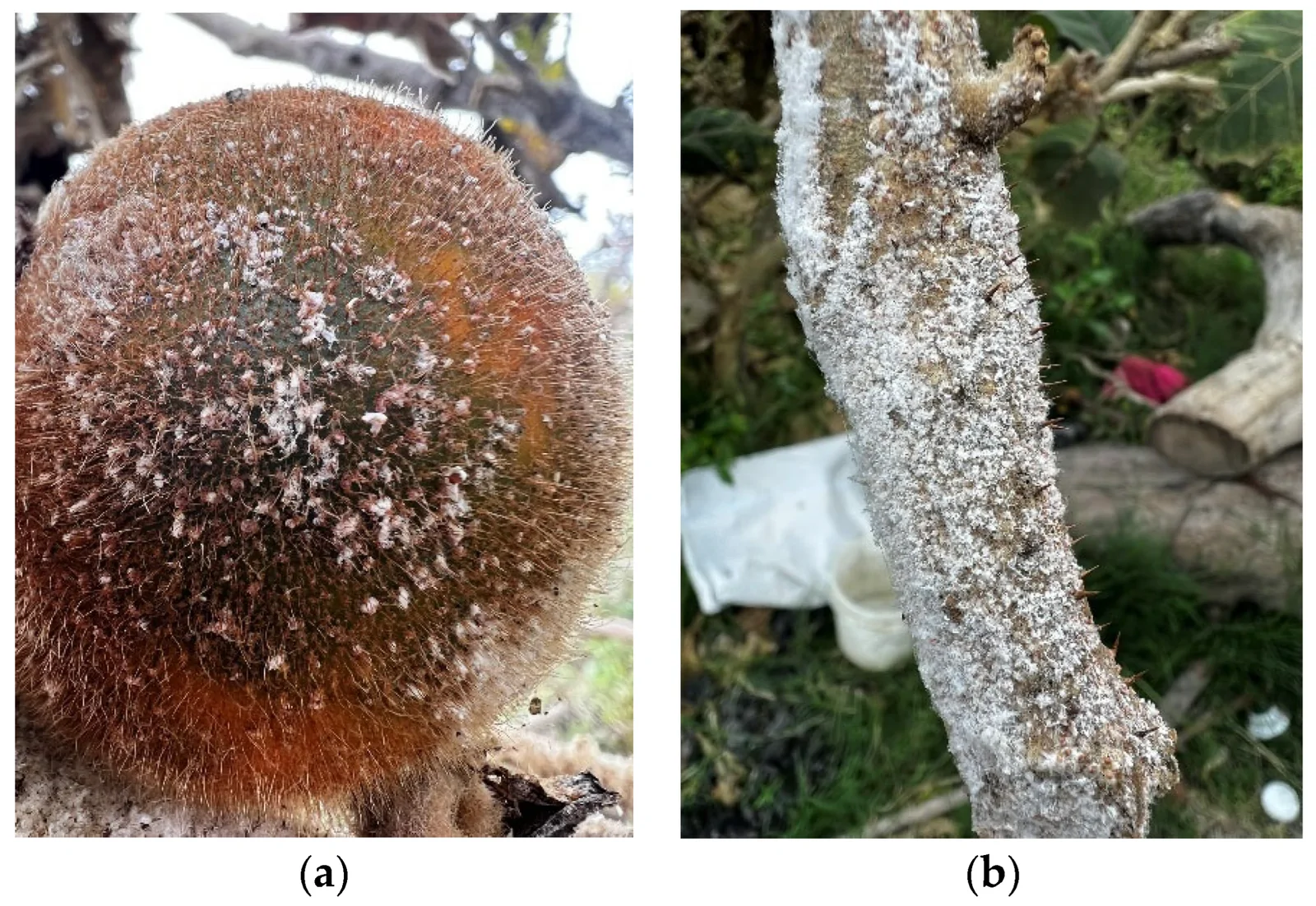
White peach scale (WPS), (**a**) *Pseudaulacaspis pentagona* on an immature fruit of *Solanum quitoense* showing indentations at the feeding site. (**b**) Heavily infested *Solanum quitoense* stem (Photo source: T.-F.B.-V.).

Infestation by *P. pentagona* causes a range of direct agronomic impacts, primarily associated with continuous sap feeding from woody and semi-woody host tissues. This trophic activity interferes with water and assimilate transport, leading to progressive plant decline, reduced vegetative vigor, defoliation, and branch dieback when populations reach high densities. In fruit crops, damage is further expressed as commercial depreciation of fruits (Figure 4), characterized by pinkish or reddish discoloration around the peduncle insertion point, which reduces visual quality and market value even when yield by weight is not immediately affected [94,113].

The negative effects of *P. pentagona* tend to accumulate over time, particularly in perennial crops, where persistent infestations lead to chronic weakening of the host plant, increased susceptibility to water stress, and reduced productive longevity of orchards. This pattern has been documented in commercial fruit crops as well as in ornamental and forest species, where the formation of dense colonies on trunks and main branches can severely compromise plant architecture and photosynthetic capacity [93,94].

From an economic perspective, impacts associated with *P. pentagona* are insufficiently quantified and show high variability depending on crop type, region, and management level. Most available studies describe damage qualitatively or through global loss estimates, without establishing standardized indicators of yield or quality reduction attributable exclusively to this pest. This limitation hinders comparisons among production systems and underscores the need for local studies integrating agronomic assessments and economic analyses [113].

Beyond direct agricultural effects, *P. pentagona* may generate indirect ecological impacts by altering interactions between host plants and their associated organism communities. In forest ecosystems and agro-urban landscapes, severe infestations can contribute to the decline and death of ornamental trees and woody species, favoring structural ecosystem changes and increasing dead biomass. Moreover, intensive insecticide use in the absence of integrated strategies can negatively affect natural enemies and other functional components of agroecosystems, amplifying ecological risks [94]. Overall, available evidence indicates that the agronomic and ecological impacts of *P. pentagona* are significant but likely underestimated, particularly in regions where comprehensive assessments are lacking.

### 3.5. Natural Enemies and Biological Control

*Pseudaulacaspis pentagona* is associated with a diverse complex of natural enemies, mainly hymenopteran parasitoids and generalist predators, which play a crucial role in regulating its populations across agroecosystems. Among the most frequently reported parasitoids are species of the genera *Encarsia* and *Aphytis*, particularly *Encarsia berlesei* and *Aphytis* spp., widely recognized for their effectiveness in suppressing diaspidid populations in fruit and woody crops [103,117].

Coccinellid predators are also key components of *P. pentagona* biological control. Species such as *Chilocorus* spp. and *Rhyzobius lophantae* have demonstrated high predation capacity on various life stages—especially nymphs and adult females—significantly reducing population densities under field conditions. Studies conducted in the Mediterranean region, particularly in Greece, highlight *Chilocorus bipustulatus* (L.) and *Rhyzobius lophanthae* Blaisdell as dominant natural enemies associated with substantial natural control rates in infested orchards [113].

Parasitism and predation rates may be high under favorable environmental conditions; however, their effectiveness is influenced by seasonality, host plant structure, refuge availability, and the presence of hyperparasitoids, which may reduce the efficiency of primary control agents [103,106]. In addition, indiscriminate use of broad-spectrum insecticides can severely affect natural-enemy survival, leading to population imbalances and pest resurgence.

Consequently, integrating conservation biological control, complemented by augmentative biological control when appropriate, represents a strategic component of integrated pest management (IPM) programs targeting *P. pentagona*. Compatibility with cultural practices and selective insecticide use is essential to enhance natural regulation and reduce reliance on chemical control [106,117]. For Ecuadorian Andean fruit production, biological control is particularly relevant due to the predominance of smallholder systems, the ecological sensitivity of mountain agroecosystems, and the need for sustainable, low-impact management strategies. Identifying, conserving, and evaluating local natural enemies therefore represents a key research priority.

## 4. Integrated Pest Management (IPM) for Andean Fruit Production in Ecuador

Integrated management of *P. pentagona* in Andean fruit systems should be based on a preventive, stepwise, and adaptive approach that combines phenological monitoring, cultural practices, conservation of natural enemies, and rational use of selective insecticides. Given the species’ multivoltine nature, temperature dependence, and the climatic heterogeneity of Ecuador’s inter-Andean valleys, IPM strategies must be finely tuned to local microclimates and host phenology, prioritizing system sustainability and phytosanitary risk reduction.

**Monitoring and early warning**

Phenological monitoring is the cornerstone of IPM for *P. pentagona*. Degree-day models using a base temperature of approximately 10.5–12.5 °C are recommended to predict crawler emergence, the most susceptible stage to control. Monitoring should be complemented with adhesive bands or sticky cards placed on branches and trunks to detect crawler dispersal peaks and optimize intervention timing, reducing application frequency and impact.

**Cultural and preventive measures**

Cultural practices play a critical role in reducing initial infestation foci. These include sanitary pruning of infested branches, safe removal and destruction of contaminated plant material, and systematic sanitation of orchards and surrounding areas. In nurseries and propagation systems, traceability schemes, regular inspections, and preventive quarantine are essential, as plant movement is a primary pathway for pest introduction and spread.

**Selective chemical control**

Insecticide use should be strategic and selective within IPM programs. Products effective against crawlers, such as buprofezin, should be applied in a targeted manner and synchronized with crawler emergence peaks identified through thermal monitoring. To minimize resistance risks, modes of action must be rotated, calendar-based applications avoided, and broad-spectrum insecticides restricted to protect beneficial entomofauna.

**Biological control and conservation**

Conserving natural enemies is central to IPM in Andean fruit systems, particularly in smallholder agriculture. Protection of parasitoids (*Encarsia*, *Aphytis*) and coccinellid predators (*Chilocorus*, *Rhyzobius*) requires careful selection of compatible inputs and provision of refuge habitats within and around orchards. Where early infestations or population imbalances are detected, pilot augmentative releases may be evaluated under local ecological conditions.

**Training and capacity building**

Successful IPM implementation depends largely on local capacity strengthening. Continuous training programs for growers, nursery operators, and technicians should focus on accurate pest identification, recognition of life stages, systematic sampling techniques, and adoption of good agricultural practices. Integrating traditional knowledge and responsible use of locally available botanical resources can further improve adoption and effectiveness of IPM strategies in Ecuadorian Andean fruit production.

Overall, implementing an IPM program tailored to Ecuadorian conditions will reduce the risk of establishment and spread of *P. pentagona*, mitigate the impact of potential infestations, and sustain productivity of Andean fruit systems in line with preventive phytosanitary protection and sustainable management principles.

## 5. Literature Review and Research Trends

Our analysis of 105 articles confirms that recent research on *P. pentagona* prioritizes distribution/invasions and predictive models, with comparatively less development in IPM and economic damage quantification. This imbalance suggests that, although predictive capacity has improved (degree-day models, niche/MaxEnt), there remains a need for integrated protocols and standardized economic metrics to support management decisions.

The concentration of studies in Asia and the Mediterranean basin, together with the low scientific output reported for the Andean region of South America, reinforces the relevance of proactive surveillance in Ecuador. The frequent mention of hosts such as Prunus, Morus, Actinidia, and Camellia sinensis in abstracts implies potential risk in inter-Andean valleys, nurseries, and agro-urban landscapes. Consequently, transferring thermal models and pheromone-based monitoring from these systems to Andean fruit production represents an immediate and cost-effective pathway.

This review has some limitations that should be acknowledged. Despite the use of multiple databases, relevant studies may have been missed due to language, accessibility, or coverage constraints. The literature is also geographically biased toward Asia and the Mediterranean region, with limited information from the tropical Andes. In addition, publication bias and the predominance of studies on invasion ecology and distribution modeling may influence the interpretation of current research trends. Nevertheless, the review provides a comprehensive synthesis of available evidence and highlights key priorities for future research and management.

## 6. Implications for Andean Fruit Production Systems in Ecuador and IPM Recommendations

### Research Gaps and Priorities

Despite sustained progress in understanding the biology, distribution, and management of *Pseudaulacaspis pentagona* internationally, critical information gaps persist that limit the formulation of effective prevention and management strategies for Andean fruit production in Ecuador. Based on the synthesis of recent literature, the following research priorities are identified as essential:

(i) Quantification of yield and quality losses

There is a widespread lack of standardized studies quantifying yield and commercial quality losses specifically attributable to *P. pentagona*, particularly in perennial fruit crops in Andean regions. Most studies describe damage qualitatively or through broad economic estimates, hindering comparisons among crops, regions, and management levels. Generating local data would enable prioritization of control actions based on realistic economic thresholds [9].

(ii) Local calibration of phenological and distribution models

Although degree-day and potential distribution models (e.g., MaxEnt) have proven effective elsewhere, their direct application in Ecuador requires local calibration and validation, considering the high heterogeneity of Andean microclimates and altitudinal effects. Model adjustment would improve crawler emergence predictions, optimize control windows, and anticipate climate-change risk scenarios [117].

(iii) Evaluation of long-term IPM programs

Most available studies assess isolated control tactics, while medium- and long-term IPM trials remain scarce. Priority should be given to evaluating programs integrating local natural enemies, cultural practices, monitoring, and selective insecticide use, accompanied by cost–benefit analyses to determine technical and economic feasibility in family farming and Andean fruit systems [9].

(iv) Varietal resistance in nationally important fruit crops

Recent advances in resistance or reduced susceptibility have focused mainly on kiwi and tea. However, similar research is needed in key Ecuadorian fruit crops such as peach (*Prunus persica*), plum, and avocado, to integrate varietal selection as a complementary IPM tool and reduce reliance on chemical measures [108].

(v) Strengthening taxonomic diagnosis

Correct taxonomic delimitation of *P. pentagona* from related species (e.g., *P. prunicola*) remains challenging in regions with high diaspidid diversity. Strengthening identification protocols through combined morphological traits and molecular markers—particularly the mitochondrial COI gene—is essential to avoid diagnostic errors that could compromise phytosanitary surveillance and timely control implementation [117].

Addressing these gaps will facilitate a shift from a reactive approach to a preventive and proactive phytosanitary management model, strengthening Ecuador’s capacity to anticipate, detect, and manage the risk associated with *P. pentagona*, while improving sustainability and resilience of Andean fruit production under climate-change scenarios [2].

## 7. Conclusions

*Pseudaulacaspis pentagona* is a highly adaptable pest characterized by pronounced polyphagy, thermal plasticity, and persistence across diverse agroecosystems, which explains its invasive success and global phytosanitary relevance. The evidence synthesized in this study demonstrates that effective management requires integrating biological and climatic knowledge with IPM strategies based on phenological monitoring via thermal models, rational and selective insecticide use, and—most critically—the conservation and enhancement of beneficial entomofauna.

In Ecuador, even in the absence of officially published records confirming its presence, the combination of climatic suitability, diversity of perennial hosts, and trade- and plant-movement dynamics justifies the adoption of a preventive and proactive approach. Early implementation of phytosanitary surveillance systems, rapid-response protocols, and early detection programs in nurseries and agro-urban landscapes is essential to reduce the risk of introduction and establishment of this quarantine pest [101,102].

This review also underscores the urgent need to generate local evidence to adapt and validate monitoring tools, phenological models, and IPM strategies to the specific conditions of inter-Andean valleys. Such efforts will not only protect Ecuadorian Andean fruit production but also reduce pesticide dependence, strengthen productive sustainability, and align phytosanitary management with the realities of family farming and agro-urban landscapes that characterize much of the national agricultural system [9].

Overall, adopting an integrated, preventive, and evidence-based approach will place Ecuador in a stronger position to confront the risk posed by *P. pentagona*, while promoting more resilient agricultural systems in the face of emerging pests and future climate-change scenarios.

### 7.1. Recommendations for Phytosanitary Public Policies

Based on the evidence synthesized in this review, the following public policy recommendations are proposed to strengthen prevention and risk management of *Pseudaulacaspis pentagona* in Ecuador, consistent with plant health principles, productive sustainability, and protection of family agriculture:

### 7.2. Strengthening Preventive Phytosanitary Surveillance

*P. pentagona* should be prioritized within national phytosanitary epidemiological surveillance programs, even in the absence of official presence reports. Surveillance should focus on ornamental and fruit nurseries, agro-urban landscapes, plant-material entry points, and wholesale markets, as these systems function as entry pathways and early reservoirs for invasive pests [101,102].

### 7.3. Rapid Response and Contingency Protocols

Public policy should include updating or developing rapid-response protocols for emerging quarantine pests, including outbreak delimitation, intensive sampling, confirmed taxonomic diagnosis (morphological and molecular), and initial management measures. These protocols would significantly reduce the economic and environmental costs associated with advanced infestations, aligning with a preventive and cost-effective approach [9].

### 7.4. Integration of Predictive Tools into Phytosanitary Management

Institutional adoption of degree-day-based phenological models and potential distribution models is recommended as decision-support tools in plant health management. Their use would allow anticipation of high-risk periods, optimization of monitoring campaigns, and targeted interventions, especially in climatically heterogeneous regions such as inter-Andean valleys [9,97].

### 7.5. Promotion of IPM as a State Policy

Agricultural policies should promote IPM as the core strategy against *P. pentagona*, prioritizing conservation of natural enemies, selective and rational insecticide use, and progressive reduction of broad-spectrum products. This approach is particularly relevant in family farming systems, where input costs and environmental risks have greater social impact [9].

### 7.6. Strengthening Technical Capacity and Training

Investment in continuous training programs for technicians, growers, and nursery operators is recommended, focusing on early identification of armored scales, systematic sampling, recognition of natural enemies, and IPM best practices. Local capacity building is key to transforming phytosanitary surveillance into a shared responsibility between the State and productive stakeholders.

### 7.7. Promotion of Territorially Focused Applied Research

Public agricultural science and technology policies should prioritize applied research lines on *P. pentagona* in Andean fruit crops, including economic impact studies, local calibration of phenological models, long-term IPM evaluations, and varietal resistance. Generating local evidence is essential to adapt global solutions to national production realities [108].

### 7.8. Interinstitutional and Regional Coordination

Finally, strengthening coordination among plant health authorities, research institutions, local governments, and producer organizations, as well as regional cooperation with neighboring countries, is recommended. Given the transboundary nature of invasive pests, regional coordination improves prevention efficiency and reduces the risk of subnational and national spread [101].

## Figures and Tables

**Table 1:** Main research categories on *P. pentagona*.

Category	Frequency	%
Geographic distribution and invasions	25	23.81
Biology and life cycle	20	19.05
Host plants	19	18.10
Prediction models	19	18.10
Chemical control	9	8.57
Biological control methods	7	6.67
Economic and agricultural impact	5	4.76
Others (general)	1	0.95

**Table 2:** Countries publishing the most on *P. pentagona.*

Region/Country	Frequency	%
China	17	16.19
Japan	14	13.33
Iran	14	13.33
United States	11	10.48
Turkey	9	8.57
Italy	5	4.76
Germany	4	3.81
Other countries (Russia, Ukraine, Greece, etc.)	31	29.52

**Table 3:** Most frequently reported hosts/crops.

Host	Frequency	%
Peach/*Prunus* spp.	56	36.84
Mulberry/*Morus* spp.	32	21.05
Tea/*Camellia sinensis*	22	14.47
Kiwifruit/Actinidia spp.	19	12.50
Ornamentals	8	5.26
Papaya/Carica papaya	5	3.29
Grapevine/Vitis vinifera	4	2.63
Olive tree (Olea europaea)	4	2.63
Persimmon (Diospyros kaki)	2	1.32

**Table 4:** Control mechanisms for *P. pentagona.*

Type of Control	Frecuency	%
Biological control	27	25.71
Chemical control	26	24.76
Monitoring	12	11.43
Integrated Pest Management (IPM)	11	10.48
Cultural control	2	1.90
Quarantine/measures	3	2.86
Not specified	24	22.86

**Table 5:** Temporal distribution of *P. pentagona* studies by decade.

Decade	Frequency	%
2020s	38	36.19
2010s	39	37.14
2000s	17	16.19
1990s	6	5.71
1980s	1	0.95
1970s	2	1.90
1960s	1	0.95
1950s	1	0.95

## Data Availability

No new data were created or analyzed in this study. Data sharing is not applicable to this article.

## Abbreviations

The following abbreviations are used in this manuscript:

EPPO	European and Mediterranean Plant Protection Organization
IPM	Integrated Pest Management
WPS	White peach scale
GBIF	Global Biodiversity Information Facility
IPCC	International Plant Protection Convention

## Appendix A

The information corresponding to the 105 scientific studies included in the systematic review and meta-synthesis of *Pseudaulacaspis pentagona* supports the thematic classification, quantitative synthesis, and analysis of research trends developed in the present study.

**Table A1:** List and characteristics of the studies included in the systematic review of *Pseudaulacaspis pentagona.*

Year	Author(s)	Characteristics
2026	Hou, H. et al.	Modern line of research in China on *Pseudaulacaspis pentagona*, focused on: Insecticide evaluation and Integrated Pest Management (IPM)
2025	Xie, W.	Local phenology, chemical control
2025	Dokuyucu, Ö. et al.	Predicting future distribution and generation number of mulberry scale under climate change in Turkiye
2025	Yin, H. et al.	Integrated risk evaluation of *Pseudaulacaspis pentagona* in China
2025	Dokuyucu, Ö. et al.	Temperature-dependent development on mulberries
2025	Jafari, M. et al.	Integration of mineral oil and washing
2025	Zhang, Y. et al.	Susceptibility of cultivars
2024	Skvarla, M. & Schneider, S.	New host records in North America
2024	Yuan, B., & Xie, W.	Life cycle (phenology)
2024	Serri, S. et al.	New record of biological control agent
2023	Zheng, X. et al.	Resistance of kiwifruit cultivars
2023	Dokuyucu, Ö. et al.	Life table at different temperatures
2023	Heikal G.A.M. et al.	Prediction of population and bioinsecticides efficacy
2023	Takahashi, A. et al.	New tea cultivar resistant
2023	Lu, Y. et al.	Species delimitation of Pseudaulacaspis
2023	Jiang, X.	Occurrence, damage and ecological control (green management)
2023	Zhang, S. et al.	The use of adjuvants, especially Luopurui, enhances the effectiveness of the insecticide.
2023	Karpun, N. & Shoshina, E.	Introduction of invasive pests through ornamental plants. Among them: *Pseudaulacaspis pentagona* (mulberry scale)
2022	Takeda, M.	Effect of changing temperatures
2022	Ozawa, A. et al.	Monitoring using pheromone traps
2022	Lit, I. L. et al.	Coccoidea infesting coconut
2022	He, R. et al.	Field trials using 6 insecticides
2022	Keskin, C. et al.	Coccoidea species and natural enemies
2021	Uchida, K. et al.	Occurrences in peaches and control
2021	Saba, T. et al.	New tea cultivar Kanaemaru
2021	Yoshida, T. et al.	Effectiveness of pesticide application
2021	Çiftci, Ü.; Bolu, H.	First records in Turkey
2020	Castro, M. T. et al.	Mulberry hosting scale insects including *Pseudaulacaspis pentagona* in Brazil
2020	Tozlu, E. et al.	Evaluation of entomopathogenic bacteria
2020	Stathas, G. J. et al.	Biology and ecology on fruit trees
2020	Lu, Y. et al.	Potential global distribution under climate change
2020	Kuzmin, R. et al.	Effect of host plant variety
2020	Toorani, A. H. et al.	Seasonal population fluctuations
2020	Ozawa, A.; Uchiyama, T.	Effects on parasitoid *Arrhenophagus*
2020	Ozawa, A.; Uchiyama, T.	Residual toxicity of insecticides
2020	Bull, B. C. et al.	Suitability as hosts for lady beetle
2020	Borzykh, A. et al.	Pheromone monitoring of orchard pests
2019	Toshiba et al.	Control effects of pesticide buprofezin on *Pseudaulacaspis*
2019	Abbasi Mojdehi, M. R.; Ghannad Amooz, S.	Population fluctuations and parasitism
2019	Toorani, A. H. & Abbasipour, H.	Parasitoids in northern Iran
2019	Stannard, K. A. et al.	Survival on kiwifruit in coolstorage
2019	Toorani, A. H. et al.	Biodiversity of parasitoids
2019	Ozawa, A.; Uchiyama, T.	Susceptibility of ladybird beetles
2019	Kakoki, S. et al.	Effect of partial pesticide spraying
2019	Cvetkovska-Gjorgievska, A. et al.	New invasive species records
2019	Wang, R.	Occurrence (population dynamics), damage and control strategies of *Pseudaulacaspis pentagona* in walnut cultivation
2019	Zou, Y. et al.	Field efficacy of six insecticides
2018	Maleki, S. et al.	Kiwifruit status in Iran
2017	Toorani, A. et al.	Report of parasitoid *Encarsia perniciosi*
2017	Toorani, A. et al.	Parasitoids in kiwi orchards
2017	Bayoumy, M. H. et al.	Geographical study and parasitoids
2017	Kyparissoudas, D. S.	Flight of males and crawler appearance
2017	Mustafaeva, G. A. A. K.	Diversity of recently recorded mealybugs including species such as *P. pentagona.*
2017	Çalışkan Keçe, A. F.; Ulusoy, M. R.	Armored scale insects on ornamentals
2016	Dalstein, M. et al.	Management of *Pseudaulacaspis pentagona* in French blackcurrant
2016	Toorani, A. et al.	First report *Aphytis chrysomphali*
2016	Mohammed, E. et al.	Distribution and natural enemies
2016	Rauleder, H. et al.	First record of predator gall midge
2016	Suh, S.-J.	Parasitoids on Prunus salicina
2016	Miramirkhani, N. et al.	First record Karnyothrips
2016	Zhuang, Q. G. et al.	Predicting phenology on kiwifruit
2015	Zhuang, Q. G. et al.	Mineral oils for control and phytotoxicity
2015	Halawa, A. M. et al.	Population fluctuation and associated natural enemies
2015	Tokumaru, S. & Yamashita, K.	Pesticide susceptibility method
2015	Paoli, F. et al.	Ultrastructure of spermiogenesis
2015	Follett, P. et al.	Release and establishment of Encarsia
2014	Abd-Rabou, S. et al.	Encarsia parasitoids in Egypt
2014	Wan, N. F., Ji, X. Y., & Jiang, J. X.	Natural enemies and biological control
2011	Rauleder, H.	Antagonists and predators
2011	Ozawa, A.	Side effects (indirect benefits) of the insecticide pyrifluquinazon applied against Pseudaulacaspis pentagona in tea plants.
2010	Li et al.	Population dynamics and spatial distribution of Pseudaulacaspis pentagona in apricot trees
2010	Neumann, G. et al.	Effect of papaya trunk angle
2010	Bazrafshan, M. et al.	Toxicity of insecticides
2010	Ozawa, A.	Insecticide susceptibility on tea
2010	Lu, C. et al.	Two new species of Septobasidium
2010	Neumann, G. et al.	Host specificity in Encarsia
2010	de León, J. H. et al.	Molecular markers for Encarsia
2009	Kozár, F. et al.	Daily rhythm of emergence and flight of males
2008	Hill, G. et al.	Control of *Pseudaulacaspis pentagona* on kiwi
2008	Kaneko, S., & Furuki, T.	Ecological relationship between the pest *Pseudaulacaspis pentagona* and one of its main natural enemies: *Pseudoscymnus hareja* (predatory ladybug)
2008	Kaneko, S., & Furuki, T.	Distribution of *Pseudoscymnus hareja* in Shizuoka tea plantations and its potential as a biological control agent against *P. pentagona*.
2008	CAB International	Pseudaulacaspis pentagona [Distribution map]
2008	Vitenko, V. A.	Forecast of the risk of mass infestations of *Pseudaulacaspis pentagona* in Ukraine
2008	Ozawa, A. et al.	Arrhenophagus albitibiae and Pseudo Scymnus hareja as key natural enemies of *P. pentagona*,
2007	Pantoja, A. et al.	Pseudaulacaspis pentagona from peaches in Puerto Rico
2007	Abbasipour, H.	Developmental time on hosts
2007	Hill, M. G. et al.	Controlling white peach scale in Italy
2006	Takeda, M.	Effect of temperature on diapause
2006	Kaneko, S. et al.	Seasonal prevalence of predator
2006	Katai, Y., & Ozawa, A.	Evaluation of different pesticide application equipment (special sprayers) and their influence on product distribution in the plant and the effectiveness of Pseudaulacaspis pentagona control
2006	Follett, P. A.	Irradiation as phytosanitary treatment
2004	Takeda, M.	Effects of temperature on oviposition
2003	Mizuta, T.	Development and reproduction on tea varieties
2001	Takashi Mizuta	Identifies resistant varieties as a control method
1997	Erkiliç, L. B.; Uygun, N.	Development time and fecundity in Turkey
1995	Zeng Xinnian	Main aspects of the peach tree white scale (Pseudaulacaspis pentagona) in relation to kiwi cultivation
1992	Zhang Xiaorui, & Chen Jian	Application in agricultural management
1992	Hanks, L. & Denno, R.	Life-history in Maryland, highly cited foundational work in the study of this species.
1991	Meyer, J.R., & Nalepa, C.A.	Effect of dormant oil treatments on white peach scale (Homoptera: Diaspididae)
1990	McLaughlin, J. R.	Chemical ecology and insect-insect communication
1983	Yasuda, S.	Influence of mulberry plant fertilization on the sex ratio (males and females)
1979	Heath, R. et al.	Identifies for the first time the sex pheromone of the female *Pseudaulacaspis pentagona*
1971	Van Duyn, J., & Murphey, M.	Life history and control of white peach scale
1965	Brown, S.W.	Systematically investigated the chromosome systems of 140 species of Diaspididae and Phoenicococcidae
1958	Bennett, F. D., & Brown, S. W.	Biological and cytogenetic research on the Pseudaulacaspis pentagona.

**Table A2:** Main hosts among food crops and ornamental species present in Ecuador with potential risk of infestation by *Pseudaulacapsis pentagona.*

Host Species	Common Name	Host Type	Main Use in Ecuador	Risk Level
*Carica papaya*	Papaya	Tropical fruit crop	Extensive commercial cultivation	***
*Mangifera indica*	Mango	Tropical fruit crop	Commercial and household cultivation	***
*Theobroma cacao*	Cocoa	Tropical fruit crop	Agroforestry/export crop	***
*Citrus* spp.	Citrus	Fruit crop	Commercial and household use	***
*Manihot esculenta*	Cassava	Food crop	Rural and subsistence farming	***
*Psidium* spp.	Guava	Fruit crop	Domestic and local use	***
*Morus alba*/*Morus nigra*	Mulberry	Fruit tree/ornamental	Fences, shade, urban areas	***
*Hibiscus rosa-sinensis*	Hibiscus	Ornamental	Gardens and urban areas	***
*Codiaeum variegatum*	Croton	Ornamental	Gardens, nurseries	***
*Catharanthus roseus*	Periwinkle/Madagascar vinca	Ornamental	Garden and pots	***
*Euphorbia pulcherrima*	Poinsettia	Ornamental	Seasonal commercial crop	***
*Nerium oleander*	Oleander	Ornamental	Living fences, urban areas	**
*Ficus carica*/*Ficus* spp.	Fig/Ficus	Fruit–ornamental	Domestic and urban use	**
*Plumeria* spp.	Frangipani	Ornamental	Tropical gardens	**
*Allamanda* spp.	Allamanda	Ornamental	Gardens	**
*Guazuma* spp.	Guácimo blanco	Native tree	Agroforestry/shade	**
*Schinus* spp.	Pepper tree	Ornamental/urban tree	Street and landscape tree	**
*Gossypium* spp.	Cotton	Crop	Limited production	**
*Cedrela* spp.	Cedar	Forest species	Reforestation/urban use	**
*Salix humboldtiana*	Native willow	Riparian tree	Wetland areas	**
*Vitis vinifera*	Grapevine	Fruit crop	Localized production	**
*Prunus* spp.	Peach, cherry	Fruit crop	Very localized production	**
*Actinidia deliciosa*	Kiwi	Fruit crop	Rare/experimental	*
Fraxinus, Ulmus, Tilia	Temperate trees	Ornamental	Very uncommon	*
Aesculus, Platanus, Paulownia	Temperate ornamentals	Urban	Very scarce presence	*

Note: *** High risk: Frequent hosts, high plant density, pest reservoirs, economic or ornamental relevance; priority for surveillance and integrated management. ** Medium risk: Common hosts but less extensive; localized impact; important as secondary infestation sources. * Low risk: Rare temperate-climate species or very limited use; low real phytosanitary impact in Ecuador. Source: [4].
